# Mediating Role of Stress at Work in the Relationship of Alexithymia and PTSD among Emergency Call Operators

**DOI:** 10.3390/ijerph182312830

**Published:** 2021-12-05

**Authors:** Małgorzata Wojciechowska, Aleksandra Jasielska, Michał Ziarko, Michał Sieński, Maciej Różewicki

**Affiliations:** 1 Department of Mother and Child Health, Poznan University of Medical Sciences, 33 Polna Street, 60-535 Poznan, Poland; malgorzata59@onet.eu; 2 Faculty of Psychology and Cognitive Science, Adam Mickiewicz University, 89AB Szamarzewskiego Street, 60-568 Poznan, Poland; ziarko@amu.edu.pl (M.Z.); sienski@amu.edu.pl (M.S.); 3 Emergency Notification Centre, 13a Wiśniowa Street, 61-477 Poznan, Poland; macroz@st.amu.edu.pl

**Keywords:** emergency call center dispatchers, stress at work, alexithymia, PTSD

## Abstract

Aim: The main purpose of this research was to investigate the relationship between alexithymia, stress at work, and post-traumatic stress disorder (PTSD) in impact emergency call center operators working in Poland (province of Greater Poland). The risk of exposure to critical life events was also considered. Methods: Data were collected using self-report questionnaires administered after dispatchers’ shifts. The emergency call center operators (N = 66) completed the Impact of Event Scale—Revised, 20-item Toronto Alexithymia Scale, Workplace Perceived Stress Questionnaire, and a questionnaire measuring the frequency and intensity of potentially traumatic events faced by emergency operators (a questionnaire developed by the authors). Results: Twenty of the most frequent events (e.g., child sexual harassment, rape, etc.) were identified. Results indicated that post-traumatic stress positively correlated with (a) work-related stress and (b) one aspect of alexithymia: difficulty expressing feelings. Additionally, work-related stress was identified as a mediator for the relation between alexithymia and the intensity of post-traumatic stress. Conclusions: The results of this study confirm that emergency operators are a high-risk group for the development of PTSD. The study results suggest that performing the work of an emergency dispatcher is not only demanding but also inherently involves participation in potentially traumatic events (as encountered through emergency calls).

## 1. Introduction

The Public-Safety Answering Point (PSAP) receives emergency calls that may include car accidents, burglaries, natural disasters, and assaults, as well as domestic violence, murder, suicide, sexual assault, etc. Operators of this service are required to obtain information about the reported incident, assist the caller, and organize on-site assistance. Despite the fact that their work is extremely emotionally demanding, this group has received no significant interest from stress researchers [1].

Public-Safety Answering Point operators are often confronted with demanding situations where the decision to provide help must be made within seconds. The caller must be supported and understood [2,3], and the emergency operators are required to actively translate callers’ excited, often frantic expressions of need into pragmatic, real-time decisions [4]. Operators are also exposed to lively, intense, and infectious caller emotions [5].

We believe that the high work demands of emergency operators, especially the uncertainty and unpredictability of reported situations, engender stress at work and expose workers to manifold traumatic events [6]. The load of stress and risk of secondary trauma exposure is much greater than that experienced in the general public [7,8,9]. Despite the lack of direct participation in a traumatic situation, they listen, respond to, and intervene in various types of traumatic events [8,10]. Their working experience is characterized by emotional difficulties that are comparable to those of emergency service workers who work on the front lines. It has been confirmed that when a person’s exposure to critical, traumatic events is potential and ubiquitous, it can lead to disorders caused by post-traumatic stress (PTSD), occupational stress, or other coexisting stress-related problems such as mental disorders [1,6,7,8,11,12,13,14,15,16,17,18,19,20]—for example, secondary traumatic stress, depression, or anxiety disorder [21]. This seems to be particularly relevant in the context of observations of the relationship of PTSD with complex, negative consequences, such as suicidal behaviors [22].

In addition, it seems justified to consider the occurrence of indirect trauma (vicarious trauma) in this group [10,23] as well as complex trauma and/or post-traumatic stress injury (PTSI) [24]. The consequences of high stress experienced in providing post-traumatic assistance [25] are defined as secondary traumatic stress disorder (STSD), also known as compassion fatigue (CF). Secondary traumatic stress disorder applies to people who are not directly exposed to a traumatic event but who express symptoms of PTSD similar to those of injury victims (e.g., therapists, emergency workers, doctors, nurses, or close relatives of victims). The effects of STSD can be compounded by insufficient social support received by emergency workers [26], which, in this industry, can manifest itself, among others, in poor communication with colleagues [27].

There are reports of research on paramedics and other emergency services, but there is a lack of data on the psychological burden on emergency operators [6]. In addition, most existing publications focus on the formal, metric aspects of work: the type of work; occupational group; demand for work during working hours; nature of a major disaster; frequency of critical or sociodemographic events such as gender, age, education, and the average age of rescue workers in assessment [8,12,16,17,28]; and there is a lack of attention in studies of emotional factors.

Existing papers on the mental health of emergency operators have many limitations, and the results they provide seem inconsistent. On the one hand, the burdens and negative consequences for mental health resulting from contact with traumatic events are highlighted in research [29]. In particular, the impact of operators’ work on the development of secondary traumatic stress disorders [11]. On the other hand, there are reports indicating that such experiences may develop emotional competence [30] or lead to post-traumatic growth [11,31]. In our research, we decided to take the first perspective. This choice is based on the belief that the negative outcomes of experiencing stress are more common than the positive consequences experienced by a few.

Based on the findings of a previous report [32], we have decided to adapt the job demands–resources model [33] on the emotional functioning of emergency call operators. That model describes how work-related stress can be explained by two basic sets of risk factors: work demands and resources. In this model, the high work demands include workload, time constraints, and a demanding environment [6]. Demands depending on the characteristics of the work may lead to physical, social, or organizational effort. For this reason, professions that require permanent mental and physical strain lead to real physical and emotional costs for employees [34,35]. Many known factors coexist with PTSD in frontline workers. These include neuroticism [36], depression [37], sense of coherence [38], ego defense mechanisms, empathy [39], resilience [40], self-efficacy [31], and are rather stable characteristics of individuals across time. For this reason, in our research, we have decided to consider a more dynamic secondary feature: alexithymia. This state may be regarded as a protective strategy—one that is reactive in nature and based on repressive coping with emotions. If the hypothesis that secondary alexithymia is a form of coping with strong negative emotions is found to be valid, it can be predicted that emergency operators experiencing more chronic work-related psychological stress will demonstrate a higher level of alexithymia [41].

The exploratory purpose of this study was to assess the frequency of experiencing traumatic/critical life events in the study group and the percentage of dispatchers with PTSD and alexithymia. The following research hypotheses were formulated:

1. Dispatchers will report higher levels of stress at work than the general population.
2. There is a positive relationship between self-reported work stress, PTSD, and alexithymia.
3. Work-related psychological stress is a mediator in the relationship between alexithymia and PTSD.

## 2. Materials and Methods

Participants: This study involved 66 emergency call operators working in the PSAP, a unit of the Emergency Information Centre in Poznań (54.5% of the total employees at the facility). A similar share of women (*n* = 35; 53%), men (*n* = 31; 47%), and transgender (*n* = 0; 0%) was included in the sample. The average age of the respondents was *M* = 31.09 (*SD* = 7.72), and years of work experience was *M* = 3.81 (*SD* = 2.46). On average, the respondents worked 43.74 (*SD* = 6.78) hours per week in their main occupation. During on-call duty, the respondents received between 100 and 210 calls (*M* = 159.84; *SD* = 27.83). In addition, 27 (40.9%) operators stated that they work in one additional place, four declared working in two places (6.1%), and one person (1.5%) declared working in three places. On average, the additional jobs took 11.38 (*SD* = 14.70) hours per week.

In addition, it was found that various demographic indicators, such as the age of respondents (*rho* = 0.038; *p* = 0.764), their length of service (*rho* = 0.090; *p* = 0.474), the number of weekly working hours (*rho* = 0.021; *p* = 0.869), and the number of answered calls (*rho* = 0.053; *p* = 0.677) did not correlate with the intensity of post-traumatic stress symptoms.

Procedure: The study was conducted in the second half of June 2018 with the approval of the dispatching institution. The persons taking part were employees of one of the local PSAPs. Applications to participate in the study were voluntary, and participants were assured of anonymity. Data were collected after completion of dispatchers’ shifts. The subjects first gave their informed consent to participate in the study and then completed the individual questionnaires. The research was questionnaire-based and conducted as a survey. The survey was conducted using the pencil–paper method and lasted, on average, 30 min.

## 3. Methods

The battery of research 4 tools included:

1. Impact of Event Scale—Revised (IES-R) [42] was used to assess the severity of PTSD symptoms. It consists of 22 statements and takes into account three dimensions of PTSD: intrusion (e.g., ‘I had difficulty sleeping all night’); avoidance (e.g., ‘I tried to avoid talking about the event’); and agitation (e.g., ‘I had difficulty concentrating’). The participant responds to statements on a 5-point Likert-type scale ranging from 0 (‘not at all’) to 4 (‘decidedly yes’). The obtained results illustrate the respondent’s subjective level of discomfort with correlation to the experienced trauma. Based on the results, groups with low and moderate/high intensities of PTSD symptoms can be identified. The limit value between the groups is 33 points (or M = 1.5 points).
2. Twenty-item Toronto Alexithymia Scale (TAS-20) [43,44] was used to measure the severity of alexithymia symptoms. The scale consists of 20 statements and 3 subscales that measure: difficulties in describing feelings/emotions (e.g., ‘It is difficult for me to find the right words to express my feelings’), difficulties in identifying feelings/emotions (e.g., ‘I am often embarrassed when I try to determine what emotions I feel’) and an operational, outward-looking style of thinking (e.g., ‘I prefer to talk to people about everyday activities rather than their feelings’). The subject responds to statements on a 5-point Likert-type scale, where ‘1’ means ‘I completely disagree’ and ‘5’ means ‘I fully agree’. In addition, according to Bagby et al. [43], the following cut-off points may be used: equal to or less than 51 means that there is no alexithymia, equal to or greater than 61 points is sufficient to make a diagnosis, and score 52 to 60 indicates a risk of alexithymia.
3. Workplace Perceived Stress Questionnaire [45]. Workplace Perceived Stress Questionnaire was used to measure work-related stress. The tool is based on Cohen’s Perceived Stress Scale [46]. The measure consists of 10 questions (e.g., ‘During the last month, how often did you feel that you did not have control over important issues in your professional life?’) to which the respondent answers on a 5-point Likert-type scale ranging from ‘never’ to ‘very often’.

Basic descriptive statistics of the measured variables and reliability coefficients of the tools used are presented in Table 1.

4. A questionnaire was developed by the author(s) for measuring the frequency and intensity of potentially traumatic events faced by emergency operators: the emergency dispatch center operatives stress load checklist. It is inspired by the Critical Incident History Questionnaire [47], which measures exposure to traumatic events among police officers [8,48]. In the final version, our questionnaire covered 20 potentially traumatic events. The questions address both the occurrence and frequency of those experiences. The items were included in the questionnaire after conducting interviews with 112 operatives. The questionnaire consisted of two parts. In the first part, we asked operators how often they dealt with a given request. The operators answered by specifying the range of submissions that they had dealt. For example, they could indicate that they dealt with a given type of report 0 times, between 1 and 9 times, between 10 and 19 times, between 20 and 50 times, and more than 50 times. In the second part, we asked operators to indicate how aggravating the submissions of a given type are. The answers follow a Likert-type format, where ‘0’ means ‘not overloading at all’ and ‘4’ means ‘extremely overloading’ (Table 2).

## 4. Results

The data analysis was preceded by multiple imputations for missing data with the Monte Carlo (EM) method. Missing data were imputed separately for each variable measured. The obtained chi-square coefficients indicated that missing data occurred randomly and that none of the subjects missed more than 2% of items. The collected data were analyzed in three steps. The first was to identify which potentially traumatic events were encountered by the participant operators in their work—and, of those, which were experienced as most burdensome. Then, simple correlation coefficients between the variables were calculated. In the third step, the hypothesis of the median role of stress in the relationship between alexithymia and post-traumatic stress was tested. Here, a mediation analysis was conducted using the method suggested by Preacher and Hayes [49]. In the mediation analysis, a resampling procedure with 10,000 repetitions was used.

Data on the frequency and burden of receiving reports of potentially traumatic events are presented in Table 2. They have been ranked from the most to the least burdensome (as experienced by the participants). In the case of 7 of 20 calls, the most frequent response was 0. The most burdensome cases were reported for incidents involving children and related to sexual abuse (e.g., child sexual harassment, rape, threat to a child’s life, child beating by adults). Operators reported that they encountered, as the most frequent (more than 50 calls): family violence between adults (78.8%), death (72.7%), as well as the caller’s self-expressions of a life-threatening caller (65.2%). The most frequent submissions were not reported as the most burdensome. They ranked eighteenth, fifteenth, and thirteenth, respectively, on the list of most burdensome submissions.

The Impact of Events Scale—Revised was used to make the diagnosis of PTSD. Here, as suggested by Creamer, Bell, and Failla [50], the conservative diagnostic cut-off for the condition (a score of 33) was used. Of the studied sample, 15% of participants presented with traumatic stress symptoms that met this criterion.

To compare work-related stress in this group to that of the general population, Student’s *t*-test for one group was conducted. The difference was significant (*t* = 11.71; *p* < 0.001). Using a comparative result from other studies [45], it was observed that dispatchers presented with higher levels of work-related stress than the general population (*M* = 15.70; *SD* = 6.69). Moreover, based on the total alexithymia score, non-alexithymia was identified in 88% of participants, possible alexithymia in 11%, and full alexithymia in only 1%.

Because the variables were normally distributed, the *rho*-Spearman correlation coefficient was used to determine the relationships between studied factors (Table 3). The results indicate that the level of post-traumatic stress was positively related to one of the alexithymia components: difficulty in describing feelings and work-related stress. Moreover, alexithymia was positively correlated with work-related stress.

**Figure 1:**
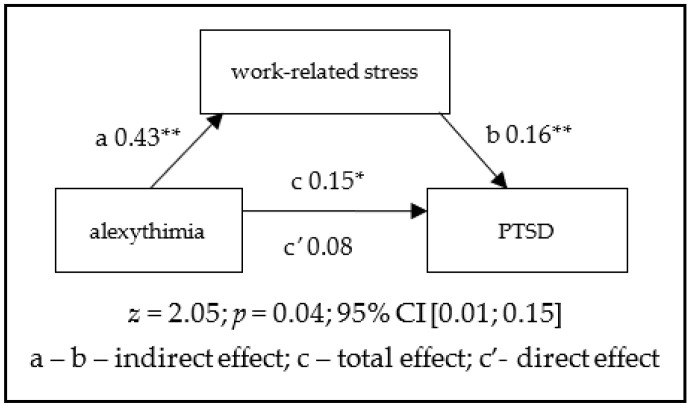
Mediation of work-related stress in the relationship between alexithymia and PTSD. * *p* < 0.01, ** *p* < 0.05.

As expected, the relationship between alexithymia and PTSD was mediated by the level of work-related stress. The observed pattern of dependency suggests that the symptoms of PTSD among 112 operators persisted, given that people with higher levels of alexithymia reported experiencing greater stress at work. Full mediation was observed (Figure 1).

## 5. Discussion

The study results confirm that emergency operators are a high-risk group for the development of PTSD, a finding that supports those of other prior studies [13,16,17,20]. In our investigation, 15% of emergency dispatchers reported symptoms that would qualify for a diagnosis of PTSD. This is similar to the results of Steinkopf and colleagues [1], who found the prevalence to be about 13–15% [51] and slightly more than in the studies by Hilaire Schneider and co-workers [10] or Kindermann et al. [21]. It is difficult to say from the tools used which type of disorder we are dealing with: primary, secondary, or complex. Due to the secondary nature of the traumatic experience, we suspect that emergency operators are more likely to develop partial (‘subliminal’) PTSD [52] or possibly post-traumatic stress injury (PTSI) [53].

Although alarm operators are often seen as less vulnerable to critical events than paramedics, they reported experiencing disturbance with such calls [6,8,10,14,48], especially those concerning child sexual abuse, rape, and threats to a child’s life. The work-related stress for this type of work [6,12,19,23,25,34,53] is higher than in the general population and may be a risk factor for physical (e.g., sleeping difficulties, and hyperarousal) and mental (e.g., burnout, and emotional exhaustion) health-related conditions that typically coexist with PTSD. The link between injury symptoms and work-related stress has also been confirmed [20,21], so PTSD can be the consequence of permanent work-related stress. These data clearly show that the work of dispatchers is overloaded in a chaotic work environment characterized by chronic stress and a lack of support [54].

The presented study has some important limitations. First of all, similar to other published studies [20,31,47], we used self-report questionnaires to estimate clinical variables. For this reason, our results are of low diagnostic value, especially for PTSD. Therefore, it is necessary to use correct diagnostics based on the DSM-5 [55]. In future studies, the use of clinical interviews to make a diagnosis should be considered. Secondly, the sample of emergency operators was not random and was highly differentiated internally; the results, therefore, may not be representative of the broader population of people in this vocation.

Additionally, the participating operators worked in the same PSAP; for this reason, the measured work-related stress might be specific to that facility. Different PSAPs may present different job demands as well as resources. These issues may limit the representativeness of the obtained results [56]. Further, the lack of a control group (nonemergency workers) or comparative group (other emergency workers) is a major limitation in our study [57,58]. In addition, assessing the frequency of exposure to various potentially traumatic events using a checklist and in a retrospective manner may limit the ability to capture other experiences not listed in the tool. In the future, researchers should consider using more accurate methods to assess exposure frequency, such as an open list or interview.

Another important point is that traumatized operators may have been on sick leave [32,59]. These issues suggest that the number of people suffering PTSD symptoms in the study group does not reflect the actual situation. The last important issue is that, to measure the stress of work, the modified version of the Perceived Stress Scale was used [46]. Although this measure presents acceptable reliability [45], it is uncertain how much the modification has negatively affected the tool’s accuracy. Although this study has some of the obvious limitations mentioned above, we believe it has the potential to significantly impact future work examining the mental functioning of emergency operators.

The outcomes of our study relate to important changes in the Diagnostic and Statistical Manual of Mental Disorders [55]. Following Carmassi and colleagues [17], the edition of the manual has better specified Criterion A by eliminating the need for a person to respond to an event involving intense fear, helplessness, or fear (AS criterion) and better explaining the characteristics of potentially traumatic experiences, including, over time, repeated, or extreme indirect exposure to aversive details of the event(s), typically during the course of work (e.g., first-aiders, and gatherers of body parts; professionals repeatedly exposed to child abuse details (A4 criterion) [11].

As we expected, the analysis results showed that in the studied group, alexithymia was not highly correlated with symptoms of PTSD. A similar effect was observed in comparable studies conducted on emergency workers [41,56]. In this context, it is difficult to clarify whether alexithymia is a risk factor for PTSD or its consequences. Similarly, it is difficult to determine whether the examined symptoms indicate the occurrence of primary or secondary alexithymia. Unfortunately, there is a lack of data on the long-term impact of work-related stress on the psychological health of emergency operators—information that could help confirm the risk of secondary alexithymia in this group. The result indicates that, in general, operators do not present with symptoms of alexithymia as a coping strategy. It turns out that personal factors such as sensitivity, empathy, intuition, and a willingness to help have a positive effect on coping with stressful situations at work [2]. Another important factor in this process is feeling pride in one’ s work [27]. Alexithymia can be considered as one of the mechanisms for dealing with stress, but it can be useful in situations of very high emotional arousal [60]. It is likely that, since emergency operators do not directly participate in traumatic events, they do not experience the emotional stimulation that would require the use of such radical emotional regulation mechanisms. It should be mentioned, however, that research reports indicate that the use of the mechanisms giving rise to alexithymia can in itself lead to experiencing higher work-related stress [61]. Operators who were considered to be performing well at work were actually coping better with stress and showed a higher level of wellbeing [31]. However, the lack of physical and visual contact with callers does not fully protect workers from emotional strain. Maintaining auditory contact is sufficient for emotional burden to occur. In this type of contact, fantasy has also been found to play a key role in regulating emotions [62].

Poverty in internal life and external thinking are characteristic of alexithymia and, as suspected, can increase work-related stress—especially in ambiguous, unclear, and confusing situations (such as when talking to an emergency line caller). The result from our study shows that the relationship between alexithymia and PTSD was mediated by perceived stress. We expected that work-related stress would act as a full mediator between alexithymia and PTSD, which could indicate the particular role of this variable in emergency operators. Results show that the alexithymic operators can experience higher work-related stress because they cannot emotionally process the stressful experiences, which predisposes them to develop PTSD. Our study, in some way, has focused on PTSD’s predictors. Low alexithymia may play a role in buffering the effect of work-related stress on PTSD. The results of our study suggest that the relationship between PTSD and alexithymia is more complex than we had previously thought. We did not find strong evidence that the postulated secondary alexithymia has a direct impact on the development of PTSD.

We suspect that any call received by an operator may become a traumatic experience. In addition, emergency workers may feel personally responsible for the outcome of their work. Their task requires a great deal of effort and can overload a worker’s mental and physical resources—and, consequently, lead to chronic stress [57]. Therefore, it is important to explore all possible important stressors: operational stressors (e.g., shift work) and organizational stressors (e.g., interactions with supervisor). The experience of emergency operators and awareness of systematic stress can form the basis for strategies to help minimize the impact of such stressors on the mental health of PSAP workers.

In addition, future research should focus on the control of intrapersonal variables, such as the operator’s emotional intelligence or emotional work, which can affect emotional expression. We also suspect that emotional regulation issues are not without significance, as they influence the reassessment of emotions experienced and may participate in stress reduction. Workplace prevention strategies can be personalized by taking into account work-related stress and work–life balance. Further research is also needed to analyze other relevant issues, such as the prevalence of PTSD and related individual differences in emergency operators, to find the most effective prevention of PTSD and other disorders associated with working in PSAP.

Since dispatchers are a particularly vulnerable occupational group to PTSD or stress in the workplace, PTSD prevention and stress-management techniques should be systematically implemented as well as attempts to reduce chronic stressors in the workplace.

## 6. Conclusions

This study revealed the presence of 20 critical events of varying frequency reported by people requesting intervention/assistance that could be potentially traumatic for dispatchers. A total of 15% of dispatchers reported having symptoms suggestive of PTSD. Post-traumatic stress disorder can be identified as a likely consequence of exposure to critical experiences at work. Work stress in dispatchers was higher than in the general population. PTSD co-occurred with work stress in approximately 14% of dispatchers. The findings related to alexithymia, although not certain and conclusive (in 12% of dispatchers, as in the general population, alexithymia was observed), provide some support for treating this feature as a nonadaptive means of processing difficult emotions. Alexithymic individuals have a higher risk of developing PTSD since they view their job as more burdensome.

## Figures and Tables

**Table 1:** Descriptive statistics, reliability coefficients, and the Kolmogorov–Smirnov test values.

Variable	Min	Max	*M*	*SD*	α	The Kolmogorov–Smirnov Test	
						*Test Value*	*p*
Post-traumatic stress PTSD total	0.00	55.00	19.70	12.18	0.88	0.12	0.024
Intrusions	0.00	15.00	5.06	3.93	0.73	0.11	0.038
Arousal	0.00	21.00	6.42	4.72	0.76	0.15	0.001
Avoidance	0.00	22.00	8.21	6.27	0.83	0.12	0.016
Alexithymia total	26.00	65.00	40.36	8.87	0.79	0.11	0.036
Difficulty describing feelings	7.00	29.00	12.54	4.88	0.80	0.18	<0.001
Difficulty identifying feeling	5.00	21.00	10.94	3.92	0.76	0.11	0.033
Externally oriented thinking	11.00	23.00	16.88	3.05	0.67	0.10	0.066
Work-related stress	11.00	40.00	26.20	7.28	0.89	0.07	0.200

α—Cronbach’s test.

**Table 2:** Frequency of traumatic events ordered by mean severity rating.

	*n*	Frequency of the Event					*M*	*Chi^2^* Test		
		0	1–9	10–19	20–50	51+		*χ^2^*	*df*	*p*
1. Child sexual harassment	66	31 (47.0%)	26 (39%)	6 (9.1%)	3 (4.5%)	0%	3.32	35.939	3	<0.001
2. Rape	66	9 (13.6%)	38 (58%)	14 (21.2%)	5 (7.6%)	0%	2.94	39.818	3	<0.001
3. The child’s life is in danger	66	3 (4.5%)	14 (21%)	13 (19.7%)	13 (19.7%)	23 (34.8%)	2.88	15.212	4	0.004
4. Adult beatings of a child	66	10 (15.2%)	33 (50%)	14 (21.2%)	6 (9.1%)	3 (4.5%)	2.85	42.333	4	<0.001
5. A multi-person accident	66	0%	3 (5%)	8 (12.1%)	16 (24.2%)	39 (59.1%)	2.68	46.121	3	<0.001
6. An accident involving children	65	1 (1.5%)	6 (9%)	13 (19.7%)	18 (27.3%)	28 (42.4%)	2.68	33.545	4	<0.001
7. Drowning	66	4 (6.1%)	36 (55%)	17 (25.8%)	5 (7.6%)	4 (6.1%)	2.62	58.394	4	<0.001
8. Being held hostage	65	45 (68.2%)	18 (27%)	2 (3.0%)	1 (1.5%)	0%	2.56	76.667	3	<0.001
9. Intention to take his own life	66	0%	7 (11%)	14 (21.2%)	16 (24.2%)	29 (43.9%)	2.36	15.333	3	0.002
10. Threats to life in hard-to-reach places	66	12 (18.2%)	23 (35%)	16 (24.2%)	8 (12.1%)	7 (10.6%)	2.30	12.939	4	0.012
11. Suicides	65	0%	10 (15%)	16 (24.2%)	18 (27.3%)	22 (33.3%)	2.28	4.545	3	0.208
12. Natural disaster	66	16 (24.2%)	17 (26%)	15 (22.7%)	0%	18 (27.3%)	1.88	3.333	3	0.504
13. The caller’s self-expressions of a life-threatening caller	65	0%	1 (1.5%)	11 (16.7%)	9 (13.6%)	43 (65.2%)	1.85	64.250	3	<0.001
14. A road accident in which a vehicle carrying a toxic substance was destroyed	66	22 (33.3%)	33 (50%)	10 (15.2%)	0%	1 (1.5%)	1.82	35.455	3	<0.001
15. Death	66	0%	3 (5%)	2 (3.0%)	13 (19.7%)	48 (72.7%)	1.80	84.667	3	<0.001
16. Fighting with serious injury	66	1 (1.5%)	13 (20%)	18 (27.3%)	12 (18.2%)	21 (31.8%)	1.67	18.000	4	0.001
17. Poisoning with a toxic substance	65	7 (10.6%)	27 (41%)	17 (25.8%)	8 (12.1%)	7 (10.6%)	1.55	23.394	4	<0.001
18. Family violence between adults	66	0%	0%	7 (10.6%)	7 (10.6%)	52 (78.8%)	1.32	61.364	2	<0.001
19. A loved one goes missing	66	0%	3 (5%)	18 (27.3%)	20 (30.3%)	24 (36.4%)	1.24	15.554	3	0.001
20. Finding a corpse	65	2 (3%)	24 (36.2%)	18 (27.3%)	10 (15.2%)	12 (18.2%)	1.17	20.970	4	<0.001

**Table 3:** Correlation matrix between variables (*rho*-Spearman).

	1	1a	1b	1c	2	2a	2b	2c
1. PTSD Total								
1a. Intrusions	0.80 **							
1b. Arousal	0.82 **	0.73 **						
1c. Avoidance	0.83 **	0.43 **	0.49 **					
2. Alexithymia total	0.21	0.18	0.24	0.16				
2a. Difficulty describing feelings	0.29 *	0.36 **	0.39 **	0.09	0.78 **			
2b. Difficulty identifying feeling	0.03	0.01	−0.02	0.09	0.79 **	0.48 **		
2c. Externally oriented thinking	0.11	−0.01	0.07	0.20	0.62 **	0.18	0.30 *	
3. Work-related stress	0.37 **	0.48 **	0.52 **	0.10	0.30 *	0.47 **	0.07	0.10

* *p* < 0.05. ** *p* < 0.01.

## Data Availability

The data presented in this study are available on request from the corresponding author. The data are not publicly available due to legal and privacy issues.
